# Influence of external calcium and thapsigargin on the uptake of polystyrene beads by the macrophage-like cell lines U937 and MH-S

**DOI:** 10.1186/2050-6511-15-16

**Published:** 2014-03-19

**Authors:** Ebru Diler, Marion Schwarz, Ruth Nickels, Michael D Menger, Christoph Beisswenger, Carola Meier, Thomas Tschernig

**Affiliations:** 1 Institute of Anatomy, Saarland University, Kirrberger Str. 100, 66424 Homburg, Saar, Germany; 2 Institute for Clinical & Experimental Surgery, Saarland University, Homburg, Saar, Germany; 3 Department of Internal Medicine V-Pulmonology, Allergology, Respiratory Intensive Care Medicine, Saarland University Hospital, Homburg, Saar, Germany

**Keywords:** Phagocytosis, Particle clearance, Macrophages, Calcium

## Abstract

**Background:**

Macrophages are equipped with several receptors for the recognition of foreign particles and pathogens. Upon binding to these receptors, particles become internalized. An interaction of particles with macrophage surface receptors is accompanied by an increase in cytosolic calcium concentration. This calcium is provided by intracellular stores and also by an influx of external calcium upon activation of the calcium channels. Nevertheless, the role of calcium in phagocytosis remains controversial. Some researchers postulate the necessity of calcium in Fc-receptor-mediated phagocytosis and a calcium-independent phagocytosis of complement opsonized particles. Others refute the need for calcium in Fc-receptor-mediated phagocytosis by macrophages.

**Methods:**

In this study, the influence of external calcium concentrations and thapsigargin on the phagocytosis of polystyrene latex beads by the macrophage-like cell lines MH-S (murine) and differentiated U937 (human) was analyzed. The phagocytosis efficiency was determined by flow cytometry and was evaluated statistically by ANOVA test and Dunett’s significance test, or ANOVA and Bonferroni’s Multiple Comparison.

**Results:**

Acquired data revealed an external calcium-independent way of internalization of non-functionalized polystyrene latex beads at free calcium concentrations ranging from 0 mM to 3 mM. The phagocytosis efficiency of the cells is not significantly decreased by a complete lack of external calcium. Furthermore, the presence of thapsigargin, known to lead to an increase of cytosolic calcium levels, did not have a significant enhancing influence on bead uptake by MH-S cells and only an enhancing effect on bead uptake by macrophage-like U937 cells at an external calcium concentration of 4 mM.

**Conclusion:**

The calcium-independent phagocytosis process and the decrease of phagocytosis efficiency in the presence of complement receptor inhibitor staurosporine lead to the assumption that besides other calcium independent receptors, complement receptors are also involved in the uptake of polystyrene beads. The comparison of the phagocytosis efficiencies of both cell types in bivalent cation-free HBSS buffer and in cell medium, leads to the conclusion that it is more likely that other media ingredients such as magnesium are of greater importance for phagocytosis of non-functionalized polystyrene beads than calcium.

## Background

The important role of macrophages in host defense against infection is known since Elie Metchnikoffs description of phagocytosis and the proposal that stimulation of phagocytes is essential for immunity [1]. Macrophages are also involved in clearance of cellular debris resulting from apoptosis or necrosis [2-4].The recognition of endogenous danger signals resulting from cell necrosis and the stimulation of macrophages by cellular debris makes macrophages one of the frontline danger sensors [5]. In general, macrophages mediate host defense, wound healing and immune regulation. Besides phagocytosis of microorganisms and erythrocytes [6], macrophages also can uptake percoll and particles, such as polystyrene beads by endocytosis, which enables the investigation of cellular and molecular mechanisms of phagocytosis [7]. This is the most important mechanism for the clearance of particles and fibers in the lung. Furthermore, particle clearance by macrophages enables targeted drug delivery to macrophages, thereby minimizing the side effects of drugs caused by systemic application. Surface functionalization of beads can maximize particle uptake e.g. by Fc-receptor-mediated uptake [8] and provides a more directed delivery to the cells. Biodegradable nanoparticles, functionalized with mannose for targeted and enhanced uptake by macrophages, which bear reactive oxygen species detoxifying catalase, were demonstrated to be scavenged by macrophages [9].

Survival of macrophages depends - to a significant extent - on Ca^2+^ influx [10]. Furthermore, Ca^2+^ is demonstrated to be essential for the efficient killing of internalized pathogens by macrophages. The inhibition of macrophage Ca^2+^ signaling by infection with *M. tuberculosis* retards the maturation of the phagolysosomes, leading to the intracellular survival of the pathogen [11]. The intracellular Ca^2+^ concentration, whether maintained by internal or external Ca^2+^ sources, was also revealed to be essential for Fc-receptor-mediated phagocytosis [12]. Incubation of some cell types with the tumor promoter thapsigargin was demonstrated to increase intracellular Ca^2+^ levels by inhibiting Ca^2+^ reuptake from the cytosol by sarco-endoplasmic reticulum ATPases [13,14].

Since data on the systemic influence of calcium on the phagocytosis efficiency of polystyrene beads by macrophages was not determined so far, the aim of this study was to analyze the influence of external Ca^2+^ concentration and thapsigargin concentration on macrophage reference cell lines. The beads were neither opsonized with complement nor functionalized with immunoglobulins. The two different cell lines - the murine alveolar macrophage cell line MH-S [15] and the activated human lymphoma cell-line U937 [16] - were used as reference cell lines. The phagocytosis efficiency was evaluated by the uptake of fluorescence dye-labeled polystyrene beads. The data revealed an external calcium-independent ingestion of polystyrene beads at physiological calcium concentrations. The phagocytosis efficiency was only slightly enhanced by a high external calcium level of 4 mM in MH-S cells, if thapsigargin was not present. Activated U937 cells showed only a significant increase in phagocytosis at an external calcium level of 4 mM if 10 nM thapsigargin was present. This cell type was not influenced by external calcium levels, if thapsigargin was not present in cell medium. Furthermore, thapsigargin did not elevate the phagocytosis efficiency in standard cell culture medium RPMI1640 with 10% FCS, FCS-free RPMI1640 and in calcium free HBSS buffer.

Understanding the mechanisms of bead uptake by macrophages is essential for the therapeutic nano- and microparticle delivery to macrophages as a potential approach for targeted drug delivery [17].

## Results

### Influence of thapsigargin concentration and medium composition on phagocytosis

**Figure 1:**
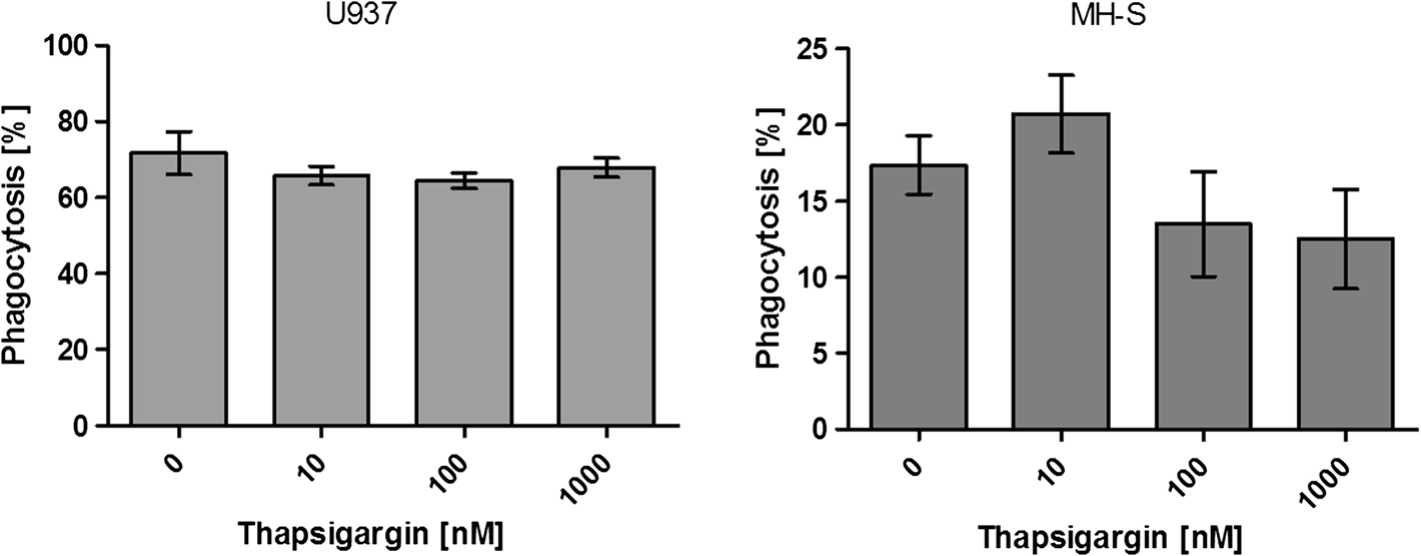
**Influence of thapsigargin concentrations from 10 nM to 1 μM on the number of phagocytic cells.** Phagocytosis efficiency was determined by FACS analysis.

The influence of the thapsigargin concentration on the phagocytosis of the fluorescent beads by differentiated (activated) U937 and MH-S cells was analyzed in RPMI1640 medium supplemented with 10% FCS. An initial number of 5 × 10^5^ cells were incubated with increasing concentrations of thapsigargin prior to the incubation with 1 × 10^7^ beads. A number of 1 × 10^4^ cells were analyzed by flow cytometry for increased fluorescence intensity caused by the uptake of particles. The graph in Figure 1 shows that a thapsigargin concentration in the range of 10 nM to 1 μM did not significantly influence the phagocytosis by U937 cells and MH-S cells (Figure 1).

**Figure 2:**
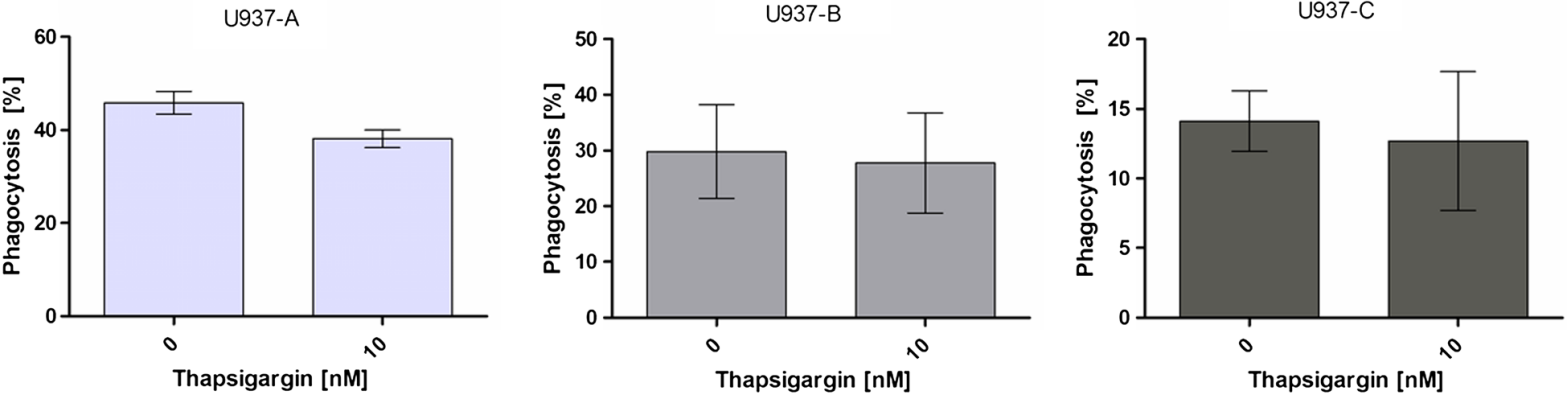
**The influence of thapsigargin on phagocytosis by differentiated U937 cells in RPMI1640 medium with 10% FCS (A), in RPMI1640 medium without FCS (B) and in HBSS buffer (C).** No significant influence by thapsigargin was determined in any medium composition.

**Figure 3:**
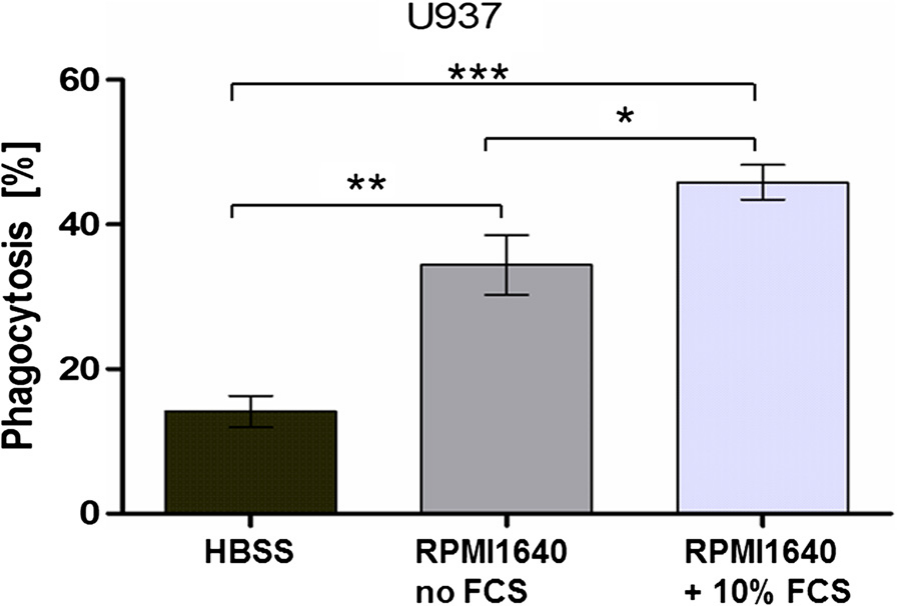
**The phagocytosis efficiency of differentiated U937 cells in different media.** HBSS buffer shows the lowest efficiency, followed by FCS-free RPMI1640 medium. Highest phagocytosis was achieved with FCS containing RPMI1640 medium.

The effect of 10 nM thapsigargin on the phagocytosis efficiency of differentiated U937 cells was investigated in RPMI1640 medium with 10% FCS (Figure 2A) in RPMI1640 medium without FCS (Figure 2B) and in external Ca^2+^-free, Mg^2+^-free and FCS-free HBSS buffer. No significant influence of 10 nM thapsigargin was determined on the phagocytosis efficiency of differentiated U937 cells in all three medium compositions (Figure 2). On the other hand, the comparison of the phagocytosis efficiencies in different media showed significant differences for phagocytosis of the beads by differentiated U937 cells. Figure 3 shows that the lowest phagocytosis efficiency was achieved in Ca^2+^-free and Mg^2+^-free HBSS buffer, followed by the value achieved in RPMI1640 medium without FCS. The highest phagocytosis rate was achieved in FCS-containing RPMI1640 medium.

**Figure 4:**
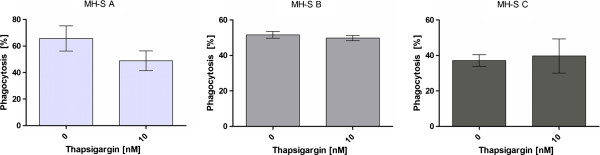
**The influence of thapsigargin on phagocytosis by MH-S cells in RPMI1640 medium with 10% FCS (A), in RPMI1640 medium without FCS (B) and in HBSS buffer (C).** No significant influence by thapsigargin was determined in any medium composition.

**Figure 5:**
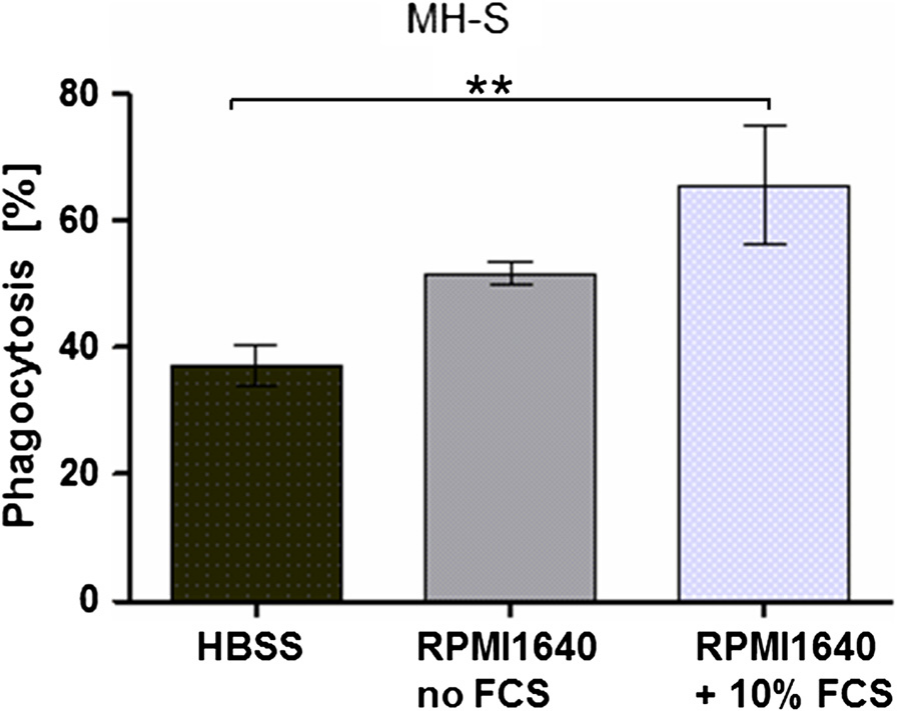
**The phagocytosis efficiency of MH-S cells in different media.** HBSS buffer shows the lowest efficiency, followed by FCS-free RPMI1640 medium. Highest phagocytosis was achieved with FCS containing RPMI1640 medium.

The concentration of 10 nM thapsigargin did not influence the phagocytosis efficiency of MH-S cells for the beads significantly in any of the medium compositions used to determine the phagocytosis efficiency in this study (Figure 4). However, in contrast to the differentiated U937 cells, the phagocytosis efficiency of MH-S cells in HBSS buffer did not significantly differ from the phagocytosis efficiency in FCS-free RPMI1640 medium. In FCS-containing RPMI1640 medium, the MH-S cells showed a significantly higher phagocytosis efficiency than in HBSS buffer or in FCS-free RPMI1640 medium (Figure 5).

### Influence of increasing external calcium ion concentrations on phagocytosis

To determine the effect of external calcium on the phagocytosis efficiency of macrophages, the reference cell lines MH-S and differentiated U937, were incubated in Ca^2+^-free, Mg^2+^-free and FCS-free HBSS buffer for one hour. Subsequently, gradually increasing concentrations of calcium chloride were added to the medium and the cells were incubated for an additional hour prior to incubation with 1 × 10^7^ beads at 37°C. To determine the influence of thapsigargin, the same phagocytosis assays were also performed in the presence of 10 nM thapsigargin.

**Figure 6:**
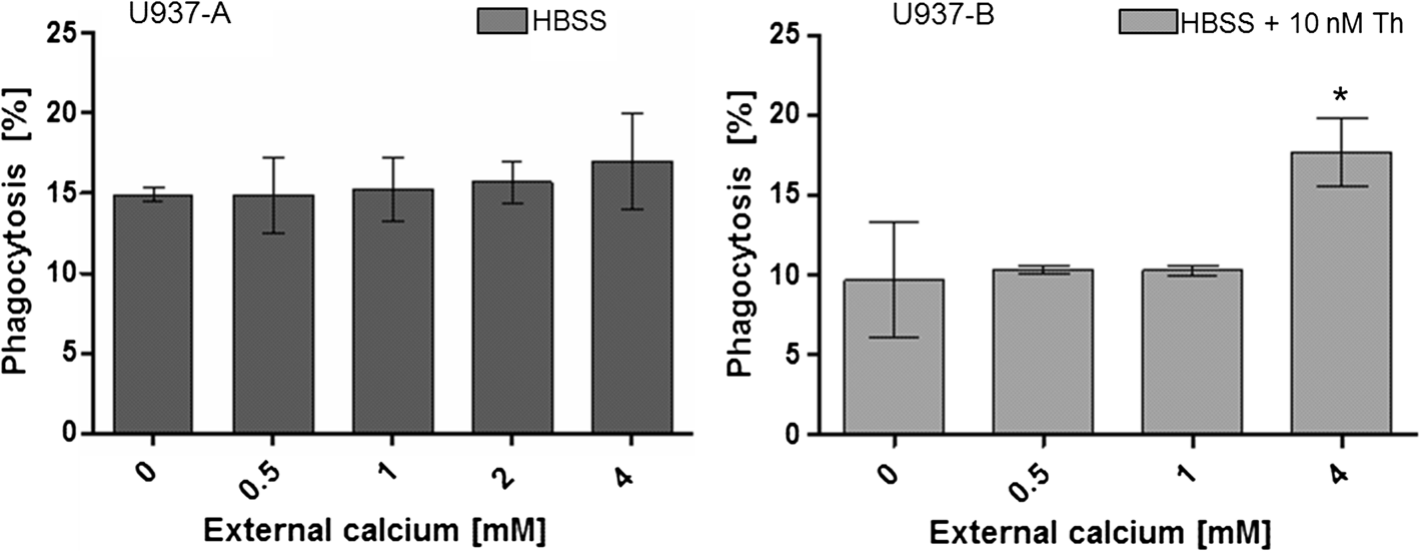
**The phagocytosis efficiency of U937 cells dependent on external calcium ion concentrations in HBSS buffer without thapsigargin (dark grey bars) and with 10 nM thapsigargin (light grey bars).** Th: thapsigargin. Statistical significance is marked by asterisks.

Figure 6 shows that external calcium did not significantly influenced the phagocytosis of beads by differentiated U937 in the range of 1 mM to 4 mM. In contrast, the presence of 10 nM thapsigargin led to a significant increase in the phagocytosis efficiency at a calcium level of 4 mM.

**Figure 7:**
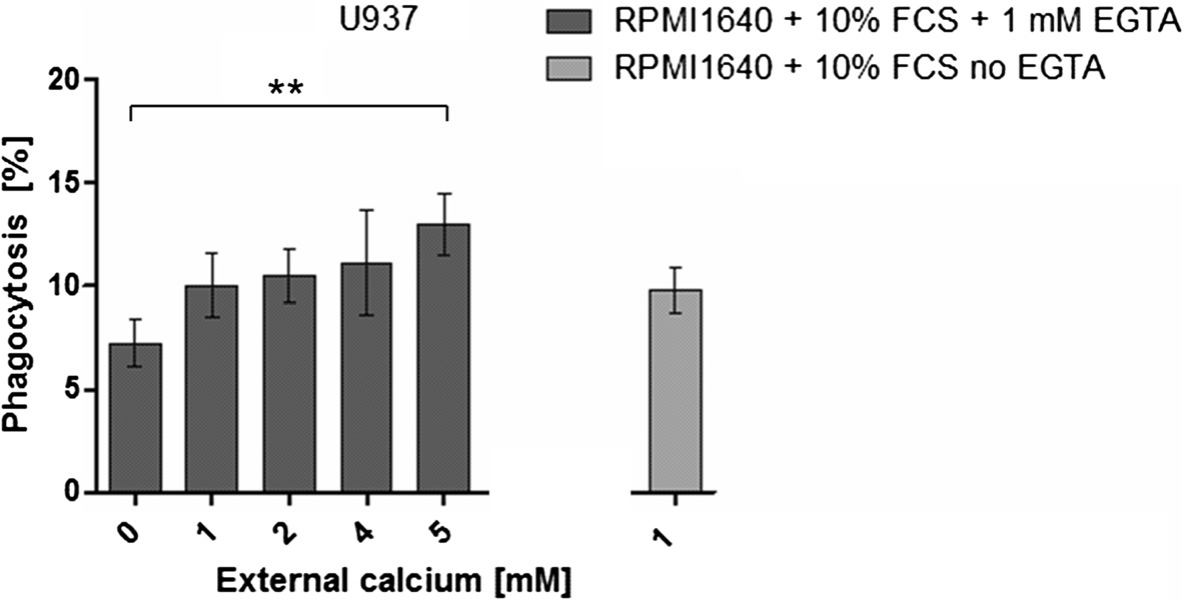
**The phagocytosis of beads by U937 cells in RPMI 1640 + 10% FCS with 1 mM EGTA and gradually increasing levels of external calcium ions (dark grey bars) compared to phagocytosis of cells incubated in EGTA free RPMI 1640 + 10% FCS with 1 mM Ca**^**2+ **^**(light grey bar).** Statistical significance is marked by an asterisk.

To exclude a decrease in the phagocytosis efficiency due to the lack of Mg^2+^ or FCS, RPMI1640 medium containing 10% FCS was incubated with 1 mM EGTA, which was demonstrated to be effective in chelating the total free Ca^2+^ in FCS-containing RPMI1640 medium (personal communication with Dr. Eva Schwarz, Saarland University Medical Center, Germany). Subsequently, increasing concentrations of CaCl_2_ were added to 1 mM EGTA containing RPMI1640 + 10% FCS medium. The EGTA-calcium complex was not removed, but as determined by the software maxchelator, the present EGTA chelates not more than 1 mM of the added external Ca^2+^ (http://maxchelator.stanford.edu/webmaxc/webmaxcE.htm). Figure 7 shows that the addition of 1, 2, and 4 mM external Ca^2+^ to 1 mM EGTA-containing medium did not enhance the phagocytosis efficiency of differentiated U937 cells for the polystyrene beads significantly. According to the calculations made by maxchelator, these external calcium values correspond to 0 mM, 1 mM, and 3 mM free calcium. These values also do not differ significantly from the phagocytosis efficiency og U937 cells in chelator-free RPMI1640 + 10%FCS. A significant increase in phagocytosis was achieved only by the addition of 5 mM Ca^2+^ to the EGTA containing medium.

**Figure 8:**
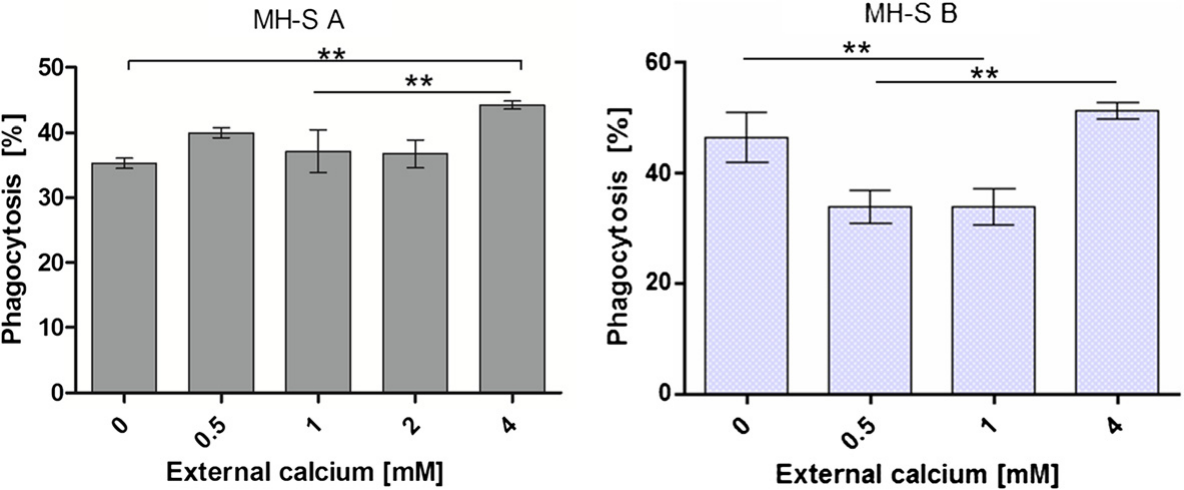
**The phagocytosis efficiency of MH-S cells dependent on external calcium ion concentrations in HBSS buffer without thapsigargin (dark grey bars) and with 10 nM thapsigargin (light grey bars).** Statistical significance is marked by asterisks.

The phagocytosis efficiency of MH-S cells were not significantly affected in HBSS buffer that contains a free Ca^2+^-concentration of 0, 0.5 mM, 1 mM and 2 mM. A significant increase in the uptake of beads by MH-S cells could be determined at a free external calcium level of with 4 mM. The presence of 10 nM thapsigargin did not significantly influence the phagocytosis efficiency. The cells showed rather a decreased uptake of the beads in 0.5 mM and 1 mM calcium containing buffer with 10 nM thapsigargin (Figure 8).

### Cytotoxicity of the medium supplements and the uptake of beads

To determine the cytotoxic effects of the media, medium supplements (thapsigargin and calcium ions) and the phagocytosis of the polystyrene beads on the differentiated U937 and MH-S cells, a toxicity assay that correlates the release of cytosolic LDH, caused by cell lysis, to the cytotoxicity of the reactants was used. A number of 1.5×10^4^ cells were used for each assay condition. The maximal level of cell lysis was determined by incubating the cells with lysis buffer leading to the lysis of 100% of the cells. These values served then as reference values for the determination of the percental cytotoxicity of RPMI1640 medium, RPMI1640 medium with 10% FCS, HBSS buffer, HBSS buffer with 5 mM Ca^2+^, HBSS buffer with 10 nM and 1 μM thapsigargin (Th). The cytotoxicity was determined for each condition in the presence and the absence of beads. The cell to beads ratio used in this assay was 1:20, according to the ratio used in the assays.

**Figure 9:**
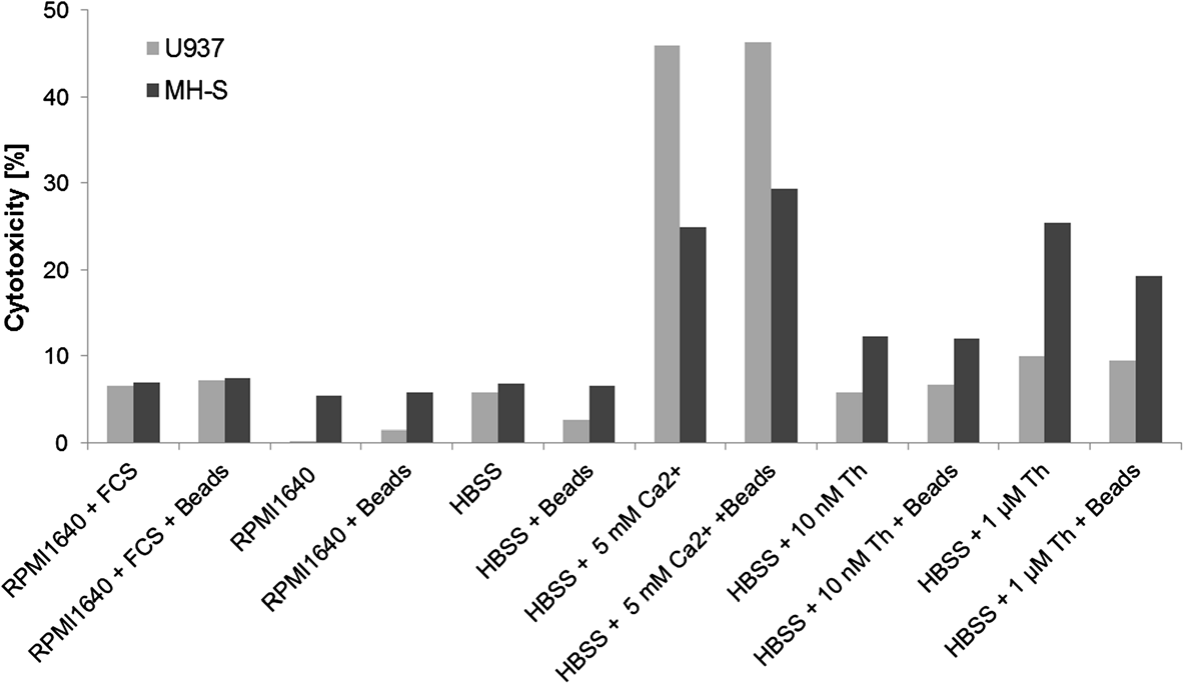
**The cytotoxic effect of medium composition, supplements and bead internalization on MH-S cells (dark grey bars) and differentiated U937 cells (light grey bars).** The cytotoxicity is determined by the activity of LHD, which is released from lysed cells. A number of 1.5 × 10^4^ cells that were completely lysed by lysis buffer served as reference.

The results of the toxicity assay for both reference cell lines can be seen in Figure 9. In general, the spontaneous lysis activity of the cells in media is less than 10% and the uptake of beads did not further increase the LDH activity, hence the phagocytosis of the beads was not cytotoxic per se. The addition of 10 nM and 1 μM thapsigargin had a greater cytotoxic effect on the MH-S cells (up to 25.5%) whereas the U937 cells remained unaffected by 10 nM thapsigargin and were only slightly affected by 1 μM thapsigargin (10% cytotoxicity). In contrast, the presence of 5 mM Ca^2+^ in HBSS buffer caused a higher cell lysis of U937 cells of up to 46%. The addition of 5 mM calcium to HBSS buffer caused the lysis of up to 29% MH-S cells.

### Determination of the adherence of beads to the cells

To verify that data obtained from the phagocytosis assays reflect the bead internalization by the cells and not only their adherence to the cells the assays were performed under two conditions that inhibit phagocytosis. However, the adherence of the beads to cells is not inhibited by these methods.

**Figure 10:**
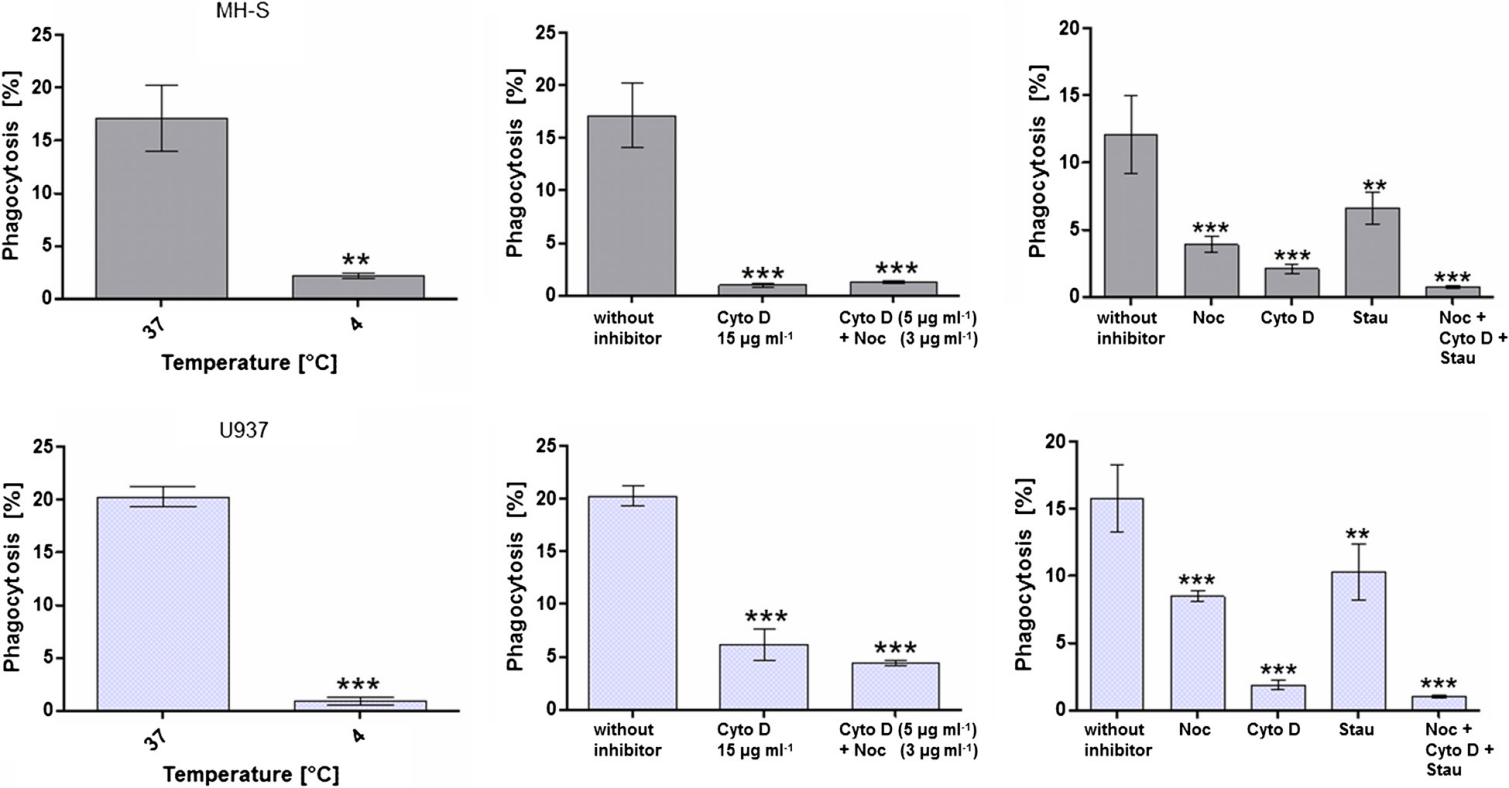
Comparison of the percentage of fluorescent cells under phagocytosis inhibiting conditions (4°C and inhibitors) with phagocytosis efficiency under non-inhibitory conditions.

First, the cells were incubated with beads at 4°C. It was previously shown that incubation of macrophages at this temperature was not lethal and did not influence the ligand binding to cells. In contrary, the ingestion of the target by the phagocytic cells is inhibited at this temperature [18]. Our data revealed that at 4°C, a significantly lower number of both type of cells were fluorescent due to bead adherence (Figure 10). This value for cells that adhered to beads (2.8% of MH-S cells and 0.9% of differentiated U937 cells) is lower than significant differences in assay conditions, where phagocytosis is performed at 37°C.

The second method for inhibiting the phagocytosis of beads was the use of three phagocytosis inhibitors: cytochalasin D (Cyto D: 5 μg ml^−1^ and 15 μg ml^−1^), nocodazole (Noc: 3 μg ml^−1^) and staurosporine (Stau: 10 μM). Additionally, a mixture of Cyto D and Noc or a mixture of Cyto D, Noc and Stau were added to the cells. The assay was performed at 37°C and the cells were incubated with the beads for 2 hours. Figure 10 shows that each inhibitor by its own caused a significant decrease in the phagocytosis of the beads but a residual phagocytosis still remained. Adding 15 μg ml^−1^ of Cyto D or a mixture of Noc and Cyto D caused a decrease in the percentage of fluorescent MH-S cells down to 0.96% and 1.3% compared to a phygocytosis efficiency of 17.13% at inhibitor-free conditions. These values were much higher for differentiated U937 cells (6.2% and 4.4%) but both cell types were strongly inhibited in their ability of ingesting beads by the simultaneous presence of all of the three inhibitors (MH-S: 0.7%, U937: 1%). These values were comparable to the results obtained from the phagocytosis assays performed at 4°C (Figure 10).

Both methods showed that the percentage of cells showing fluorescence due to bead adherence is much lesser than significant differences achieved in phagocytosis efficiencies due to different conditions. Therefore, adherence of beads did not falsify the results.

## Discussion and conclusion

Macrophages and neutrophils are professional phagocytosis cells of the innate immune system. The uptake of large particles (>0.5 μm) occurs in an actin-dependent manner. It has been demonstrated that many cells, such as bladder and thyroid epithelial cells, also accomplish phagocytosis [19] but professional phagocytes, like macrophages, possess phagocytic receptors that increase the target particle range and the phagocytosis rate [20]. The mechanisms underlying phagocytosis by macrophages are complex and provide important tools for the essential role of the cells in the uptake and degradation of infectious agents, senescent cells, particles, tissue remodeling, immune response and inflammation [21].

Macrophages are equipped with receptors for Fc-mediated, complement-mediated and pathogenic conserved motifs-mediated phagocytosis [21]. Although ligand binding to all of these receptors promotes phagocytosis, the effects of receptor signaling differ. Whereas FcR-mediated phagocytosis results in the production and secretion of proinflammatory mediators like arachidionic acid and reactive oxygen species, complement receptor (CR)-mediated phagocytosis does not cause the release of these mediators [22,23]. Interaction of ligands with phagocytic receptors for immunoglobulins (FcγR) and for complement proteins induces the activation of phospholipase C and phospholipase D, which in turn leads to the formation of Ca^2+^ mobilizing second messengers. This, on the other hand, is involved in the activation of store-operated calcium entry (SOCE) channels in the plasma membrane, resulting in a Ca^2+^ flux from the extracellular medium. It is generally accepted that an increase in cytosolic Ca^2+^ is an early signal that accompanies phagocytic ingestion, but the elevation of cytososlic Ca^2+^ is required for the promotion of an efficient ingestion of foreign particles by some, but not all, receptors. In 1985 Lew et al. showed that antibody/FcR generated phagocytosis relies on an increase of cytosolic Ca^2+^ concentration upon receptor activation, whereas activation of the complement receptor C3b/bi generates a Ca^2+^-independent phagocytosis mechanism in human neutrophils. They determined a decrease in Fc-receptor-mediated phagocytosis after increasing the intracellular Ca^2+^-buffering capacity of quin2, which was more pronounced in the absence of extracellular calcium. In contrast, a decrease in complement-mediated phagocytosis of yeast cells was not observed despite increasing the intracellular Ca^2+^ buffering by quin2 [24]. Nevertheless, the question of the necessity of Ca^2+^ for the phagocytosis of foreign particles is controversial [25]. Some studies in murine macrophages verified a decrease of phagocytic ingestion of IgG opsonized or non-opsonized latex beads due to Ca^2+^ chelation [26-28], whereas others reported effective phagocytosis of IgG opsonized red blood cells at low cytosolic Ca^2+^ levels [29-31].

In this study, the influence of external calcium levels and thapsigargin concentrations were investigated with regard to the phagocytosis efficiency for non-IgG functionalized, fluorescence-labeled polystyrene beads. The phagocytosis assays were applied on two different reference cell lines: the murine alveolar macrophage derived MH-S [15] and human lymphoma monocyte like cell line U937 [16], which were differentiated to adherent, phagocytosis-exhibiting macrophage-like cells by PMA, previous to performing phagocytosis assays.

The data acquired from thapsigargin containing assay set-ups, external Ca^2+^ free conditions, and a stepwise increase in external Ca^2+^ concentrations revealed that external calcium concentrations of 1 mM and 2 mM did not influence the uptake of non-functionalized beads by differentiated U937 cells and MH-S cells in HBSS buffer. Furthermore, neither the presence nor the absence of thapsigargin also affected the phagocytosis efficiency of these cells at physiological calcium levels. The phagocytosis efficiency of differentiated U937 cells was not even affected in their phagocytosis efficiency at an external calcium level of 4 mM. This situation changed if 10 nM thapsigargin was present in HBSS buffer. Whereas external free calcium concentrations of 1 mM and 2 mM were still ineffective in increasing the phagocytosis efficiency of differentiated U937 cells, the presence of thapsigargin enhanced phagocytosis efficiency from 9.6% to 17.7% at an external calcium concentration of 4 mM. The cytotoxicity assay showed that the increased calcium levels in HBSS buffer caused a high level of cell death of up to 46%, which was probably caused either by high osmotic pressure or by an increase in intracellular calcium levels, known to cause the death of cells [32]. To exclude decreased phagocytosis efficiency due to the lack of FCS or Mg^2+^, phagocytosis assays with differentiated U937 cells were performed in 1 m EGTA containing RPMI1640 + 10% FCS medium. This concentration of EGTA was shown to be effective in chelating the complete free calcium of the FCS containing medium (personal communication with Dr. Eva Schwarz, Saarland University). The phagocytosis efficiency of differentiated U937 cells in this medium did not show a significant difference to the phagocytosis efficiency in EGTA-free RPMI1640 + 10% FCS medium. Further increase of external calcium only showed significant enhancement in phagocytosis efficiency at a calcium concentration of 5 mM, which according to the maxchelator software, corresponds to 4 mM free calcium. But, the amount of free calcium in FCS is not known, and probably the calcium level is much higher than 4 mM. This data also provided a hint to the insignificant role of physiological calcium levels on phagocytosis.

MH-S cells showed some differences in phagocytosis efficiency at an external calcium concentration of 4 mM. In the absence of thapsigargin, the presence of 4 mM external free calcium in HBSS buffer increased the phagocytosis efficiency of MH-S cells significantly from 35% to 44%, whereas in the presence of 10 nM thapsigargin, no enhancement of the phagocytosis efficiency of MH-S cells was determined. Nevertheless, neither cell line showed any differences in their phagocytosis efficiency in the range of free external calcium at the range of 0 mM to 2 mM in HBSS buffer. We can conclude that, at physiological free calcium levels, which, according to free calcium measurements of the sera of healthy individuals, is at the range of 1.175 mM – 1.375 mM [33], the efficiency of phagocytosis of beads by MH-S cells and differentiated U937 cells is not affected by calcium.

It was previously demonstrated that the tumor promoter thapsigargin increases intracellular free Ca^2+^-levels by inhibiting the Ca^2+^ reuptake from the cytosol into the ER-lumen by sarco-endoplasmic reticulum ATPases [13,14]. In this study, experimental set-ups with increasing thapsigargin concentrations in RPMI1640 + 10% FCS revealed that the phagocytosis behavior of differentiated U937 cells and MH-S cells is not affected by the presence of thapsigargin, even at concentrations of 1 μM. As mentioned, a concentration of 10 nM thapsigargin was only effective in enhancing the phagocytosis efficiency of U937 cells in HBSS buffer with 4 mM calcium, but not at lower extracellular calcium levels. According to the cytotoxicity assay, differentiated U937 cells seem to be resistant to the presence of thapsigargin even at high concentrations. MH-S cells, on the other hand, were highly sensitive to the presence of thapsigargin, leading to a cell lysis of up to 25.5%.

The uptake of beads by differentiated U937 cells in Ca^2+^-free, Mg^2+^-free HBSS buffer was low, compared to the phagocytosis efficiency in FCS-free RPMI 1640 medium, which, according to the manufacturer, contains 1 mM free Ca^2+^. However, after verifying that this external Ca^2+^ concentration does not have an increasing effect on phagocytosis efficiency, it can be concluded that other supplements such as magnesium, which is also not present in HBSS, could have a more pronounced influence on the phagocytosis efficiency of these cells. In fact, the suppression of phagocytosis caused by lowered extracellular levels of magnesium was previously demonstrated in rat alveolar macrophages [34,35].

All of the data acquired in this study indicate that the uptake of non-functionalized polystyrene beads with a size of 1 μm by MH-S cells and by differentiated U937 cells does not depend on external calcium at physiological levels or on the addition of thapsigargin. The beads used in this study, were not opsonized with antibodies or with complement proteins but the latter type of opsonization cannot be excluded in phagocytosis assays performed in FCS-containing medium. As mentioned above, complement-mediated phagocytosis was demonstrated to be Ca^2+^ independent in human neutrophils [24]. Therefore, a complement-mediated, Ca^2+^- independent phagocytosis of polystyrene beads is a possible way of bead uptake by the reference macrophage-like cell lines used. This conclusion is further supported by the results obtained from phagocytosis studies performed in the presence of staurosporine. The uptake of beads by both cell types was significantly reduced when the complement receptor inhibitor staurosporine was added to the medium. Therefore, the calcium- independent phagocytosis process and the influence of staurosporine on bead uptake reveal a possible complement receptor-dependent way of polystyrene bead entry. However, taking into account that staurosporine did not completely inhibit the bead uptake, interaction of beads with other receptors could also cause the uptake of beads in an Ca^2+^-independent manner.

In conclusion, a comparison of the particle uptake by differentiated U937 cells and MH-S cells in Ca^2+^-containing HBSS with that in FCS-free RPMI 1640 medium indicated that other PRMI1640 ingredients, for example magnesium ions, could be more necessary for an efficient phagocytosis than free external calcium ions. Experiments to reveal the influence of external magnesium are in preparation and should shed some light onto the mechanisms involved in the uptake of polystyrene beads by macrophage-like U937 cells and MH-S cells.

## Methods

### Cells

The murine alveolar macrophages cell line MH-S was obtained from Sigma-Aldrich (Germany). These cells were grown in RPMI 1640 medium (Lonza, Germany) containing 10% heat inactivated fetal bovine serum (PAA, Germany), 100 Units ml^−1^ penicillin, 100 μg ml^−1^ streptomycin (both antibiotics from PAA, Germany) and 50 μM β- mercaptoethanol (gibco life technologies, Darmstadt, Germany) at 37°C in a humidified atmosphere with 5% CO_2_. To detach the cells from culture flasks a few milliliters of a mixture of 500 μg ml^−1^ trypsin with 220 μg ml^−1^ EDTA were added to the adherent cells, which were then incubated at 37°C for fifteen minutes. Cells were then collected by centrifugation and resuspended in medium. Then 0.5 × 10^6^ or 1 × 10^6^ cells were disseminated on 12 well-plates (Greiner Bio-One, Frickenhausen, Germany). After 15 hours the cells were used for the phagocytosis assay.

The monocyte-like human lymphoma U937 cell line was cultivated in RPMI 1640 medium with 10% fetal bovine serum, 100 Units ml^−1^ penicillin and 100 μg ml^−1^ streptomycin. For differentiation into adherent macrophage-like cells capable of phagocytosis, cells were incubated with 50 ng ml^−1^ phorbol 12-myristate 13-acetate (PMA) (Sigma Aldrich) for 20 hours, washed with medium once, and then incubated in medium for 48 hours at 37°C in a humidified atmosphere with 5% CO_2._ Subsequently the cells were used for the phagocytosis assay.

### Phagocytosis of fluorescent beads

Fluoresbrite® Yellow Green Microspheres (Polysciences GmbH, Eppenheim, Germany) with a size of 1 μm were used for phagocytosis assays. 0.5 × 10^6^ or 1 × 10^6^ cells were incubated with 1 × 10^7^ or 2 × 10^7^ beads (20× excess of beads), respectively, for 2 hours.

To determine the thapsigargin influence on the efficiency of phagocytosis, 5 × 10^5^ cells were incubated with 100 nM, 400 nM, 1 μM, and 10 μM thapsigargin for 1 h previous to phagocytosis. Then, 1 × 10^7^ beads were added to the cells and incubated for 2 hours.

External Ca^2+^ influence was determined by using Ca^2+^ free and Mg^2+^ free Hank’s Balanced Salt Solution (HBSS). After growth of the cells in RPMI 1640 with 10% FCS, the medium was removed, the cells were washed with phosphate-buffered saline (PBS) and then incubated in HBSS buffer with a stepwise increase in the concentration of CaCl_2_ rising from 0.5 - 5 mM CaCl_2_ with or without 10 nM thapsigargin for one hour previous to bead addition. The phagocytosis efficiency was analyzed by flow cytometry.

To decrease the level of free Ca^2+^ in RPMI 1640 medium containing 10% FCS, the medium was incubated with sterile filtered 1 mM ethylenglycol tetraacetic acid (EGTA). A number of 5 × 10^5^ cells per well were incubated in EGTA containing medium for 20 minutes and then increasing concentrations of CaCl_2_ from 0.5 mM to 5 mM were added stepwise to the cells. After an incubation period of 30 minutes, 1 × 10^7^ beads with a size of 1 μm were added and the cells were incubated with beads for 2 hours in a humidified incubator at 37°C with 5% CO_2_. Subsequently, the bead-containing medium was removed and cells were washed with PBS prior to trypsinization. The cells were fixed with 4% paraformaldehyde solution in PBS pH 7.4 and stored at 4°C for several days until analysis by flow cytometry.

### Flow cytometric analysis of phagocytosis efficiency

The phagocytosis efficiencies were determined by using a BD FACScan™ flow cytometer (Becton- Dickinson, Heidelberg, Germany ). Point plotting of the events measured in the fluorescence channel (ordinate) versus total events measured in the forward scatter channel (abscissa) revealed the number of cells that internalized fluorescent beads. MH-S cells and differentiated U937 cells that were not incubated with fluorescent beads were used to determine the auto fluorescence and to define the dot plot panel sizes. The phagocytosis efficiency of cells was defined as the percentage of cells that internalized beads with reference to the total cells analyzed by flow cytometry. The number of cells analyzed was between 2 × 10^3^ and 1 × 10^4^. The number of beads internalized by each cell was not considered in the determination of the efficiency of phagocytosis.

### LDH cytotoxicity assay

To determine the cytotoxic effects of different media compositions (RPMI1640, RPMI1640 + 10%FCS and HBSS) and supplements (5 mM Ca^2+^, 10 nM thapsigargin, and 1 μM thapsigargin), the Pierce™ LDH Cytotoxicity Assay Kit from Thermo Scientific (Schwerte, Germany) was used. To determine cytotoxic effect of internalization of polystyrene beads, the assay was performed in the absence and also in the presence of beads (beads to cell ratio: 20:1). The assay was performed according to the manufacturer’s instructions for chemical compound mediated cytotoxicity. Fluorescence of media compositions and LDH activity in FCS was determined in a cell-free manner and subtracted from sample results. However, in contrast to the instructions, spontaneous LDH activity was not subtracted from the sample results, because, besides toxic effect of calcium, thapsigargin and uptake of beads, the aim was also to determine the lytic effect of media compositions per se.

### Determination of adhesion of beads to the cells

The portion of bead adherence to the macrophage-like cells was determined by incubating cells and beads to a ratio of 1:20 in RPMI1640 + 10% FCS medium at 4°C. After two hours of incubation, the cells were washed with ice-cold PBS and then trypsinized. After fixation, 1 × 10^4^ cells were analyzed by flow cytometer (FACSCalibur, Becton- Dickinson, Heidelberg, Germany). The second method for determining adherence of beads to the macrophage-like cells used was the addition of phagocytosis inhibitors to the cell medium previous to phagocytosis. For this purposes, the cells were incubated in RPMI1640 + 10% FCS medium with cytochalasin D (5 μg ml^−1^ or 15 μg ml^−1^), nocodazole (3 μg ml^−1^) or staurosporin (10 μM). Furthermore, a mixture of cytochalasin D (5 μg ml^−1^) and nocodazole or a mixture of cytochalasin D (5 μg ml^−1^), nocodazole (3 μg ml^−1^) and staurosporine (10 μM) were added to the medium. The cells were incubated in inhibitor containing medium for two hours at 37°C. Subsequently the polystyrene beads were added and phagocytosis was accomplished for two hours at 37°C. A number of 1 × 10^4^ cells were analyzed by flow cytometer.

### Statistics

Calculations, graphical illustration of data and statistical analysis were accomplished with GraphPad Prism (GraphPad Software Inc., La Jolla, USA). Data are presented as mean ± standard deviation. The significance in mean value differences (P < 0.05) were determined by *t*-test and analysis of variance (ANOVA) with the post-hoc analysis of Dunnett’s or Bonferroni’s Multiple Comparison Test and labeled with asterisks.

## Abbreviations

ATPase: Adenylpyrophosphatase, Adenosine Triphosphatase; CR: Complement receptor; Cyto D: Cytochalasine D; EDTA: Ethylenediaminetetraacetic acid; EGTA: Ethylene glycol tetraacetic acid; ER: Endoplasmic reticulum; FCS: Fetal calf serum; HBSS: Hank’s Balanced Salt Solution; MH-S: Murine alveolar macrophage cell line; Noc: Nocodazole; PBS: Phosphate buffered saline; PMA: Phorbol-12-myristat-13-acetat; Stau: Staurosporine; U937: Human monocyte like cell line.

## Competing interests

The authors declare that they have no competing interests.

## Authors’ contributions

ED and TT conceived the study and conducted the experiments. MS and RN were responsible for the cell culture, performed the phagocytosis assays, flow cytometry and data analysis. CB, MM and CM participated in the study design and were involved in the interpretation of the results as well as in the drafting of the manuscript. ED participated in all parts of the study and wrote the manuscript. All authors read and approved the final manuscript.

## Pre-publication history

The pre-publication history for this paper can be accessed here:

http://www.biomedcentral.com/2050-6511/15/16/prepub
